# Mn-doped nickeltitanate (Ni_1−*x*_Mn_*x*_TiO_3_) as a promising support material for PdSn electrocatalysts for methanol oxidation in alkaline media

**DOI:** 10.1039/d1ra02883a

**Published:** 2021-08-26

**Authors:** R. Saraswathy, R. Suman, P. Malin Bruntha, D. Khanna, V. Chellasamy

**Affiliations:** Velammal Institute of Technology Panchetti Chennai 612 204 India; Department of Chemistry, Francis Xavier Engineering College Tirunelveli 627 003 India; Department of Electronics and Communication Engineering, Karunya Institute of Technology and Sciences Coimbatore 641 114 India; Department of Applied Physics, Karunya Institute of Technology and Sciences Coimbatore 641 114 India; Centre for Nanoscience and Technology, Pondicherry University R.V. Nagar, Kalapet Puducherry India 605 014 chellasamy.v@gmail.com

## Abstract

Nickeltitanate (Ilmenite) has been prepared with stoichiometric variation by substituting Mn in the ‘A’ site, using the sol–gel method in a highly active form. The PdSn electrocatalyst was then impregnated with nickeltitanate by a microwave-assisted polyol method. The physiochemical characterisation of the synthesized electrocatalyst PdSn–Ni_1−*x*_Mn_*x*_TiO_3_ was done by X-ray diffractometry, UV-visible spectrophotometry, Raman spectroscopy and transmission electron microscopy. The elemental composition was obtained using energy dispersive spectra which confirmed the presence of Ni, Mn, Ti, O, Pd and Sn. Electrochemical characterization using cyclic voltammetry and polarization experiments showed that the synthesized PdSn–Ni_1−*x*_Mn_*x*_TiO_3_ exhibited an enhanced catalytic activity and better stability in the alkaline medium, compared to conventional PdSn/C catalysts. It was observed that the charge transfers from the support material (Ni_1−*x*_Mn_*x*_TiO_3_) to the PdSn electrocatalyst boosted the oxidation reaction. By varying the methanol concentration from 0.5 M to 2.0 M, the resulting current density also varied from 129 to 151 mA cm^−2^. This result demonstrated that the prepared material PdSn–Ni_1−*x*_Mn_*x*_TiO_3_/C electrocatalyst is an excellent candidate for the methanol oxidation reaction.

## Abstract 2

Nickeltitanate (Ilmenite) has been prepared with stoichiometric variation by substituting Mn in the ‘A’ site, using the sol–gel method in a highly active form.

## Graphical abstract

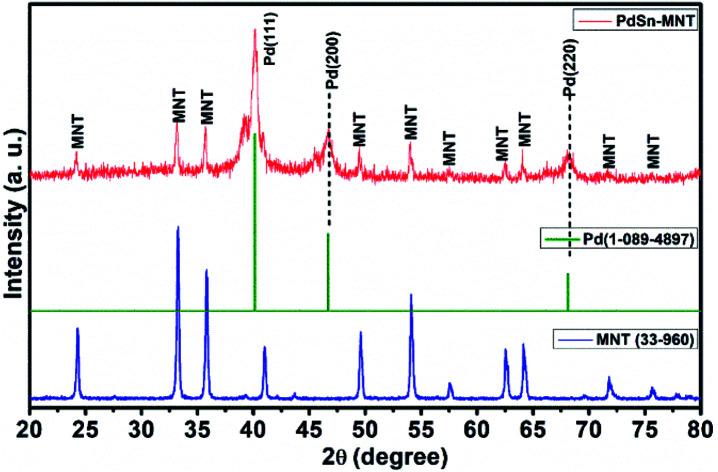

## 1. Introduction

Energy plays an essential part in the development of human civilization. Scientists and technologists are facing two problems: meeting the fast-growing demand for energy and reducing the pollution to the environment that results from the burning of fossil fuels. Various efforts are in progress all over the world to develop advanced energy systems that are eco-friendly such as fuel cells, lithium-ion batteries and photoelectrochemical systems that could provide unlimited energy supply.^1^ Small organic molecules like ethylene glycol, glycerol, formaldehyde and ethanol have gained considerable traction because of their small size which can be used in the Proton Exchange Membrane Fuel Cell (PEMFC).

Direct methanol fuel cell is a suitable system for energy conversion, because of that pollution-free process and high efficiency in energy conversion system.^2^ However, there are still some obstacles limiting the large-scale applications of PEMFC. The main problems are low electrocatalytic activity and easy deactivation of catalysts with regards to molecular oxidation. The noble metals platinum, gold and ruthenium are commonly used as electrocatalysts in fuel cells, among which palladium-based catalyst is the most cost-effective choice and relatively abundant on the earth.^3^ These noble metals lose their performance while being used as catalysts in the fuel cell for a longer period because Nafion is unstable during methanol oxidation in a fuel cell. This peculiar problem was attributed to the action of hydroxyl and hydroperoxyl free radicals on the Nafion.^4–6^

In the direct methanol fuel cell, activated carbon is used as support material, whereas commercially Vulcan carbon XC-72R is in use, because the latter one has high active surface area, more stability and high charge transfer. However, the self-agglomeration and deterioration behaviour of activated carbon leads to reduce the efficiency of the charge transfer kinetics between the metal particles and the support materials resulting in the decrease of performance of the fuel cell.^7–10^ To avoid the deterioration of activated carbon in electrocatalyst, a support material based on metal oxides have been used for the electro-oxidation reaction.^11–14^ The metal oxide support for the electrocatalyst materials has a significant role in oxidation reaction and they have a large surface area and high oxygen vacancy on the surface of the oxide materials. They have a particularly good electrical conductivity that confirms better electron transfer and high stability in the fuel cell operating environment during electro-oxidation of methanol.^15^ There are many metal oxides titanium oxide, cerium oxide, zinc oxide, tungsten oxide, iron oxide, nitrogen-doped nickel oxide and nickeltitanate being used as a support material for an electrocatalyst^16–21^

Among these materials, researchers have turned their attention to NiTiO_3_ which has high corrosion resistance in the acidic environment of a direct fuel cell.^22^ Although NiTiO_3_ showed extremely good effects on the stability and catalytic activity, the electronic conductivity of NiTiO_3_ at low temperatures inhibits its effective application in DMFC. A direct electronic transition between the higher level of the O_2_ 2p valance band and the lower level of the Ti 3d conduction band happens as the bandgap energy of the metal oxide NiTiO_3_ is 2.2 eV. The optical band gap of NiTiO_3_ is tuned by using dopants such as Ag, Mo and non-metal doped metal oxides.^23,24^ The cobalt-doped NiTiO_3_ materials has resulted in the decrease of optical bandgap from 2.34 eV for pure NiTiO_3_ to 1.91 eV for ten percentage of cobalt doping.^25^ Thus, the bandgap tuned metal oxides have been used as a support material for an electrocatalyst. However, the cobalt oxide having low stability, p-type conductivity, which is kinetically unfavourable to support fast electron transport required by high power density.^26^

Similar studies have already done earlier focused on enhancing the catalytic activity of metal oxide supported electrodes for methanol oxidation.^27^ In addition to that, the decoration of NiTiO_3_ to Pt/C was found to increase the catalytic action and stability for methanol oxidation reaction and also to prevent CO poisoning in a direct methanol fuel cell (DMFC).^28^

Manganese has multiple oxidation states such as Mn^2+^ to Mn^4+^. Hagemann in 1978 reported that transition metals like Mn-doped BaTiO_3_ were associated with charged oxygen vacancies that create ‘axial’ defects in perovskite cell Ti^4+^.^29^ And also manganese shows a large potential window, moderate conductivity, high stability, and good electrochemical reversibility.^30^ However, to the best of the author's knowledge, the applicability of Mn-doped NiTiO_3_ as a suitable support material for an electrocatalyst in DMFC hitherto is not reported.

In this present study, various characterization methods such as XRD, UV, Raman spectroscopy, and TEM were used for the analysis of crystal structure, bandgap calculation, vibration mode of support materials and the microscopic images of the prepared electrocatalyst respectively. PdSn binary nanoparticles have been adorned over the Mn-doped NiTiO_3_ nanoparticles, and the resulting nanocomposite electrocatalyst has been compared with PdSn/C in terms of electrochemical study for the Methanol Oxidation Reaction (MOR) by using cyclic voltammetry, chronoamperometry and polarization studies.

## 2. Experimental methods

An electrocatalyst has been prepared in two-step methods, first, the surface-active Ni_1−*x*_Mn_*x*_TiO_3_ materials were prepared, secondly, the prepared materials were used as active support for palladium stannous binary electrocatalyst.

The Ni_1−*x*_Mn_*x*_TiO_3_ surface-active material has been synthesized by the sol–gel method. The precursors used: nickelnitrate from Himedia chemicals for nickel source, titanium isopropoxide from Sigma-Aldrich for titanium, and manganese acetate from Himedia chemicals for manganese. All these chemicals were used without further purification. Citric acid from Fisher chemicals was used as the chelating agent and ethylene glycol from Himedia chemicals was used as a solvent. The synthesis procedure was given in our previously reported literature.^31^

The prepared Ni_1−*x*_Mn_*x*_TiO_3_ was dispersed in ethylene glycol solution and sonicated for 30 minutes using an ultrasonicator (Citizen, 50 W/40 kHz) for getting homogeneous dispersion in the solutions. The ratio of palladium and Sn ratio was 1 : 1 and the PdSn and Ni_1−*x*_Mn_*x*_TiO_3_ proportion was maintained at (1 : 1). After the addition of Pd and Sn precursors into the Ni_1−*x*_Mn_*x*_TiO_3_, the dispersed solution was stirred well and then, 0.8 M NaOH solution was added drop wisely into the above-said mixture, the pH was maintained at 9.0 and further stirred for one hour to complete the reaction. The final solution was heated in a microwave oven (LG-MH-6549QS, power 100%) for 40 seconds and cooled down naturally at room temperature. The precipitated solution was rinsed with acetone several times, and the end prepared material was dried at 70 °C for five hours and it was used for further characterization.

The crystallographic analysis was carried out by X-ray diffractometer, Ultima-IV (Rigaku) operated at 1.2 kW. The Cu-Kα1 radiation was used with the Kα filter. The scan range was 20 to 80° in Bragg–Brentano geometry. The absorption properties of the catalyst were studied by UV-vis spectrometer (Shimadzu Uv3600+). The Raman analysis was done by using Renishaw Invia system which has a laser excitation wavelength of about 785 nm using 1% of 100 mW (with 30 s exposure time). Transmission electron microscopy was taken by TECNAI-G2 operated at 300 kV and the TEM specimens were prepared as per the standard method for powder materials. The synthesized electrocatalyst was coated over a carbon film that has been pre-coated with the copper grid. The electrochemical experiments were carried out using the Electrochemical Workstation (Potentiostat Model Number: Solartron 1287) at 25 °C which is the measuring temperature (room temperature). The electrochemical impedance behaviour of the prepared electrocatalyst was taken by using Solartron 1260A impedance analyzer at room temperature. The electrochemical measurements were measured with a conventional three-electrode cell kit.

## 3. Results and discussion

### 3.1. XRD

**Fig. 1:**
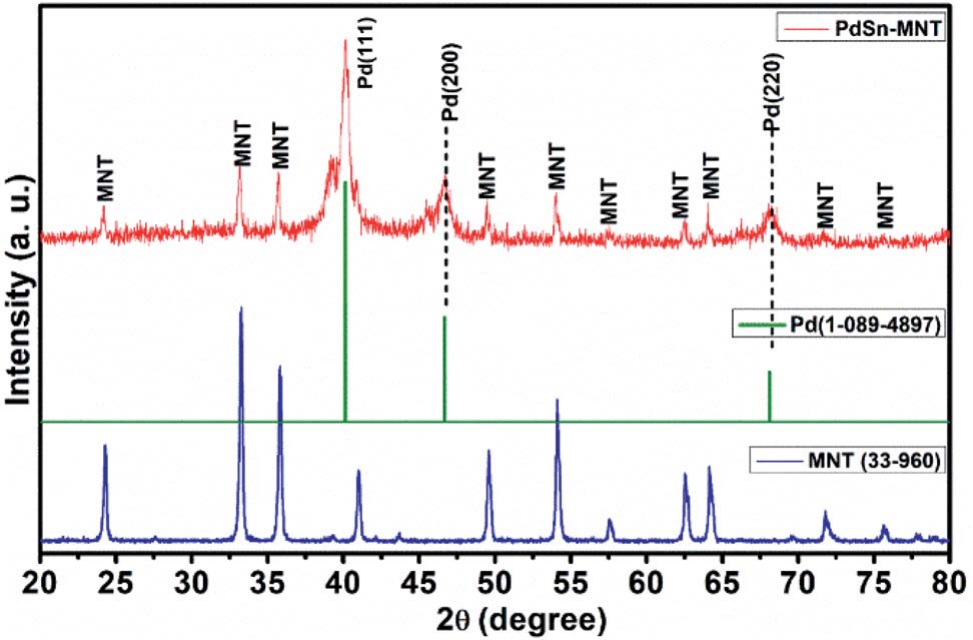
X-ray Diffraction pattern for PdSn adorned Ni_1−*x*_Mn_*x*_TiO_3_ electrocatalyst.

The crystallite and structural informations were analyzed by using the X-ray diffractometer. Fig. 1 shows the X-ray diffraction pattern of manganese doped nickeltitanate supported PdSn electrocatalyst. The diffracted peaks were attributed to the rhombohedral structured ilmenite phase of Ni_1−*x*_Mn_*x*_TiO_3_ and the peaks were matched with the ICDD data with the card number 33-960 and the synthesized electrocatalyst was marked as MNT. In addition to the Ni_1−*x*_Mn_*x*_TiO_3_ phase, three peaks were also observed in the plane of (111), (200) and (220), these peaks are corresponding to the cubic structure of the PdSn phase which has been indexed as Pd in Fig. 1, and it was compared with ICDD data card number 01-088-2335. The crystallite size was found from the indexed graph using the Debye–Scherrer equation,*D* = *kλ*/*β* cos *θ*where *D* is the crystallite size (nm), *k* is a constant, *λ* is the wavelength of the X-ray (Å), *β* is the full-width half maxima of the peak, *θ* is the Bragg angle. The crystallite size for MNT and PdSn were 56 nm and 13 nm respectively.

### 3.2. UV-visible spectroscopy of PdSn–Ni_1−*x*_Mn_*x*_TiO_3_

**Fig. 2:**
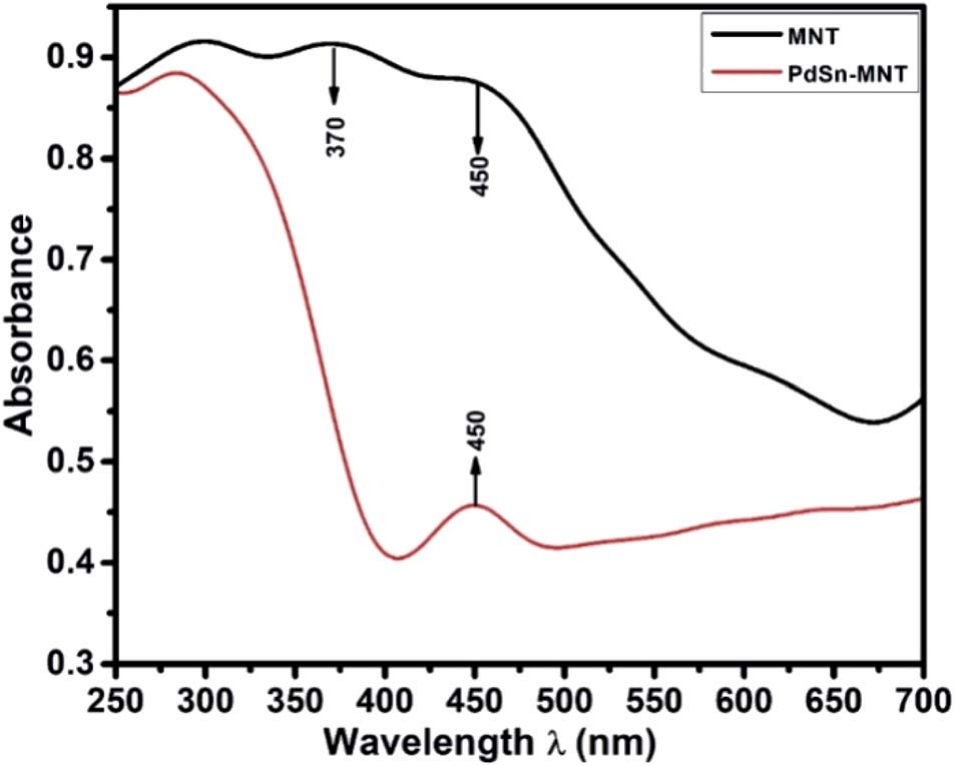
The UV-visible absorption spectrum of nanostructured PdSn–Ni_1−*x*_Mn_*x*_TiO_3_ and Ni_1−*x*_Mn_*x*_TiO_3_.

Fig. 2, UV-visible absorption spectrum depicts the optical absorbance graph of Mn-doped NiTiO_3_ (MNT) and PdSn–MNT nanocatalyst with an irradiated wavelength in the range of 250 to 700 nm. There were two absorption peaks observed for MNT at 370 and 450 nm and one active absorption peak for MNT at 450 nm, which is attributed to Ni 3d ↔ Ti 3d. According to Agui and Mizumaki,^32^ there are three types of electronic charge transfer occurring in NiTiO_3_, namely the transition between Ni 2p ↔ Ti 3d, Ni 3d ↔ Ti 3d and Ni 3d ↔ 2p.

The optical bandgap of NiTiO_3_ is 2.34 eV. Manganese-doped nickeltitanate has a lower bandgap of 1.95 eV compared to pure NiTiO_3_. This is due to the introduction of dopant manganese in the metal oxide matrix which produces defects on the surface of the metal oxides and hence a change in bandgap energy.^33^ The bandgap energy was calculated from the Tauc plot^34^ as given in the equation.*α* = *A*(*hϑ* − *E*_g_)^*n*^/*hϑ*where *α* is the absorption coefficient, *A* is a constant, *E*_g_ is the absorption band, *hϑ* ‘*h*’ is the photon energy, ‘*ϑ*’ is frequency and ‘*n*’ depends on the transition.^35^ This change in the energy gap could enhance the charge transfer between the metal and metal oxide electrode interaction.

### 3.3. Raman spectroscopy

**Fig. 3:**
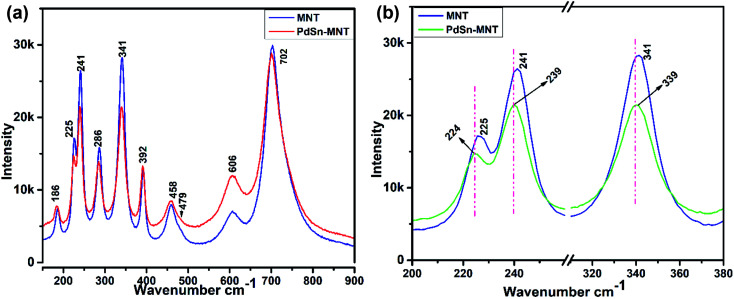
(a) Raman spectrum for the nanostructured for PdSn–Ni_1−*x*_Mn_*x*_TiO_3_ and Ni_1−*x*_Mn_*x*_TiO_3_ (b) magnified representation of selected E_g_ and A_g_ vibration modes.

Fig. 3a represents the Raman spectra for the nanostructured PdSn–Ni_1−*x*_Mn_*x*_TiO_3_ and Ni_1−*x*_Mn_*x*_TiO_3_. Whereas, the selected E_g_ and A_g_ vibration modes were magnified and presented in Fig. 3b. The electrocatalyst support material Mn-doped NiTiO_3_ has got ilmenite structure with *R*3̄ space group symmetry. The oxygen atoms are located at *x*, *y*, *z* coordination. Moreover, the nickel and titanium are occupied at (0, 0, *z*) position and the Ni atom has an octahedron position which shares a face with the nearest Ti atom in an octahedral position. The oxygen atoms have been arranged as hcp and the Ni atoms occupy 2/3rd of the octahedral sites.^36^ According to the factor group theory, NiTiO_3_ (ilmenite structured) preserves the centre for symmetry and holds the soundness of the mutual exclusion principle. One can expect ten Raman active modes in this case^37,38^ (5A_g_ + 5E_g_) and eight IR active modes (4A_u_ + 4E_u_) with the acoustic modes and has no inactive modes. In Fig. 3a, ten Raman modes at 186, 225, 241, 286, 341, 392, 458, 479, 606 and 702 cm^−1^ were witnessed which are in concordance with the previous report of Busca *et al.*

In the literature,^11^ Raman mode assignment has been carried out in two ways namely, by assessing the relative intensities of the Raman mode between parallel and cross polarisations or by comparing the Raman spectra of materials having the same structures such as CoTiO_3_ and MgTiO_3_ (ref. 39) Assignment of these Raman modes, in the present study, was carried out based on the evaluation of the experimental spectra of materials having similar structures with the values reported in ref. 10–12. The strongest mode appears at 702 cm^−1^ from the symmetric stretching of TiO_6_ octahedra. The vibrational peaks at 606 and 458 cm^−1^ are of E_g_ type. These peaks are ascribed to the twist and asymmetric breathing of the oxygen octahedra with cationic vibrations parallel to the *XY* plane. The Raman mode at 225, 286 and 341 cm^−1^ are of E_g_ type. These are attributed to the asymmetric breathing vibration of the oxygen octahedra and twist of oxygen octahedra which are due to the vibrations of Ni and Ti atoms parallel to the *X* and *Y* planes respectively. The A_g_ Raman peak at 186 cm^−1^ is allocated to the symmetric stretching vibrations of Ti–O. The other A_g_ Raman modes at 241 cm^−1^ are assigned to the vibration of Ti atoms along the *Z*-axis. The other two A_g_ modes at 392 and 479 cm^−1^ are ascribed to a breathing-like stretching of the Ti centred oxygen octahedra.

Fig. 3b evidenced the slight shift of Raman active modes to lower frequencies that could remarkably explain the influence of ‘Mn’ ions in the lattice vibration mode. The shifted Raman modes in PdSn–MNT sample inferred a strong relation with the distorted structure because of PdSn anchoring on MNT.^33^

### 3.4. Transmission Electron Microscope (TEM)

**Fig. 4:**
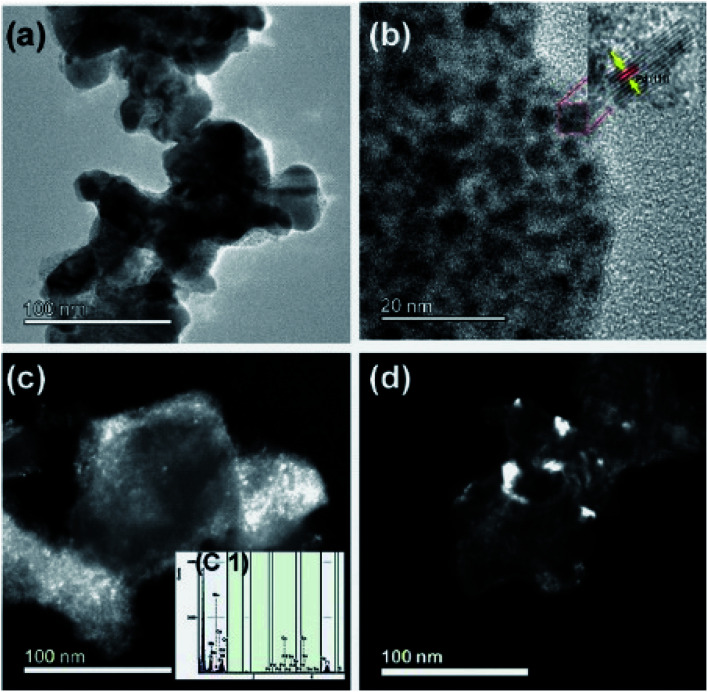
(a) and (b) Bright-field transmission electron micrographs of PdSn–Ni_1−*x*_Mn_*x*_TiO_3_, and high-resolution image inserted into image (b), (c) and (d) are the dark field images and the inserted figure in (c) is the XEDS spectrum of PdSn–Ni_1−*x*_Mn_*x*_TiO_3_.

The transmission electron microscope was utilized to study the microstructure of the electrocatalyst. In the binary electrocatalyst of palladium, tin was adorned on Ni_1−*x*_Mn_*x*_TiO_3_ metal oxides. Fig. 4a and b are the bright-field images of PdSn–Ni_1−*x*_Mn_*x*_TiO_3._ In Fig. 4b, a set of planes is marked and the interplanar spacing for that set of planes was measured to be 0.23 nm that corresponds to (111) plane of Pd(Sn) particles (cubic phase of Pd). Fig. 4c and d are the dark field images of metal oxide supported nanocatalyst and these images clearly show that the bigger particles are manganese doped metal oxides and the smaller particles are PdSn electrocatalyst.

The inserted figure in Fig. 4c is the XEDS spectrum of PdSn–Ni_1−*x*_Mn_*x*_TiO_3_ which confirms that the constituents are Ni, Mn, Ti, O, Pd, and Sn. This XEDS spectrum is of the small sample region, as the area hit by the electron beam in TEM was small. Hence the interpretations from these results are highly local.

**Fig. 5:**
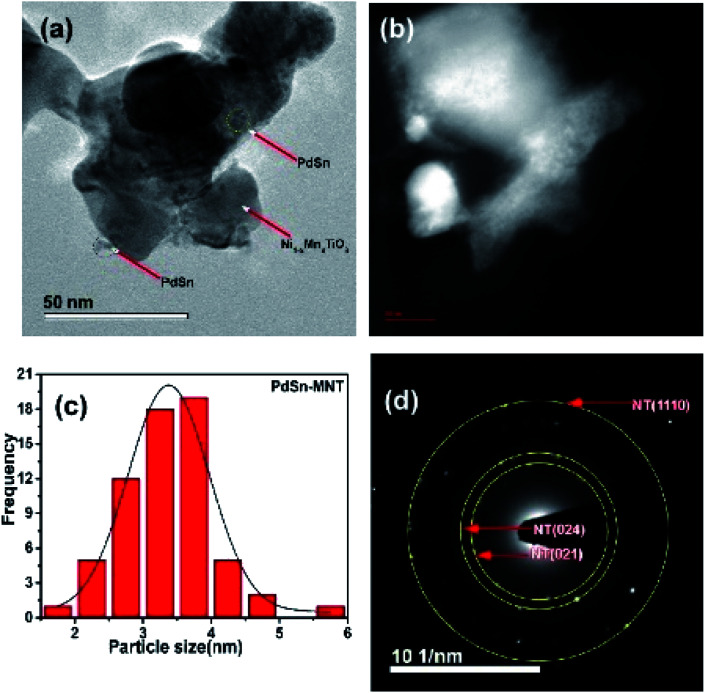
Transmission electron micrograph of PdSn–Ni_1−*x*_Mn_*x*_TiO_3_ (a) bright field image (b) STEM image of PdSn–Ni_1−*x*_Mn_*x*_TiO_3_ (c) particle distribution and (d) SAED pattern presented.

Another microscopic image (Fig. 5a) clearly shows that there are two types of particles marked as a catalyst and support materials. The decorated catalyst is the tiny particle marked as PdSn (3–4 nm) and the support material is marked as Ni_1−*x*_Mn_*x*_TiO_3_. Fig. 5b is the STEM image of PdSn–Ni_1−*x*_Mn_*x*_TiO_3_ and it shows the smaller particles decorated over the more substantial sized particles. The Pd(Sn) particles have relatively a broad distribution with a median particle size of 3–4 nm which is represented in Fig. 5c. The SAED pattern is presented in Fig. 5d. There are three planes marked for Ni_1−*x*_Mn_*x*_TiO_3_ as NT (021), (024), (110).

### 3.5. Electrochemical study

#### 3.5.1 Electrode material preparations

The modified working electrode was prepared as follows: 4 mg of the as-prepared electrocatalyst and 6 mg of activated carbon were dispersed in 150 microliters of isopropyl alcohol. 10 microliters of 0.5% Nafion solution was added into the mixture and the mixture was sonicated for 50 minutes to get a homogenized electrocatalyst. A slurry was obtained. An aliquot of five microlitres from the obtained slurry was dropped over a glassy carbon electrode and dried at room temperature. The electrochemical experiments were made in a three-electrode cell system. The working electrode was a modified glassy carbon electrode.

#### 3.5.2 Cyclic voltammetry and chronoamperometry

**Fig. 6:**
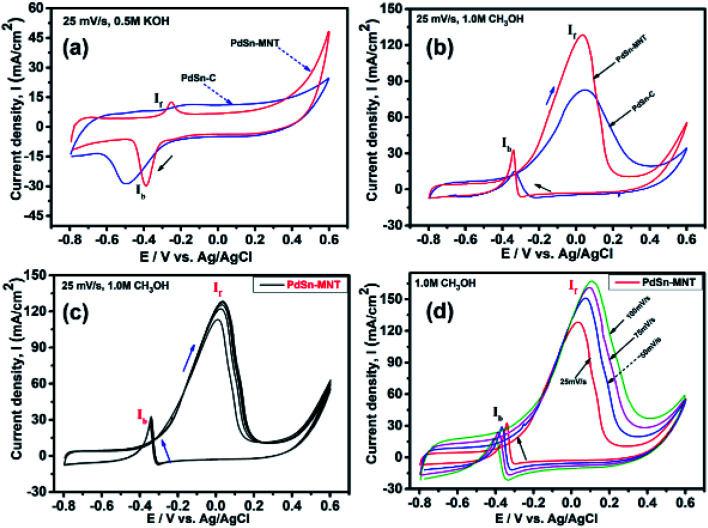
Cyclic voltammograms of nanostructured electrocatalyst (a) CV curves in 0.5 M KOH at a scan rate of 25 mV s^−1^ (b) CV curves of in 0.5 M KOH with 1.0 M methanol (c) CV curves of PdSn–MNT/C in 0.5 M KOH + 1.0 M methanol (d) different scan rate of electrocatalyst PdSn–MNT/C, all the measurements were done at a scan rate of 25 mV s^−1^.

The electrochemical analysis of the catalysts was carried out by using the cyclic voltammetry method. The CV recorded for PdSn/C and PdSn–MNT/C electrodes in alkali solutions in absence of and presence of methanol are shown in Fig. 6. The oxidation peaks appear during the forward and backward scanning due to the electro-oxidation of methanol. Before the actual performance of the experiments, fifteen successive cycles were run to stabilize the working electrode.^40^

The electrochemical performance of methanol oxidation was analyzed by the forward peak current density (*I*_f_), and the backward current density (*I*_b_) and the ratio of the *I*_f_ and *I*_b_ that is the maximum current density has been calculated from the CV curve^41^ in Fig. 6. The onset potential of PdSn–MNT/C is lower than that of PdSn/C which confirmed that methanol oxidation of PdSn–MNT/C is better than that of conventional PdSn/C. In Fig. 6b, two peaks were observed. One peak was found in the forward scan and the other peak was in the reverse scan for both the catalysts. The progressive scan of the maximum oxidation potential of the PdSn–MNT peak at 36 mV and the maximum current density of 128 mA cm^−2^ are attributed to the methanol oxidation. The backward scan is ascribed to the oxidation of the residual carbonaceous species. This was not completely oxidized in the forward scan. The homemade PdSn/C electrocatalyst has been tested for comparison. The current density is maximum (83 mA cm^−2^) at 50 mV. The cyclic voltammetry with varying scan rate was applied to investigate the reaction mechanism of the methanol oxidation on PdSn–MNT/C electrode. The oxidation potential of PdSn–MNT is 36 mV and that of PdSn/C is 50 mV. This confirmed that PdSn–MNT/C has got low polarization and higher current density during the electro-oxidation reaction. These results plotted in Fig. 6d shows that the peak current density and peak potential for the methanol oxidation becomes more prominent as the scan rate was increased. The forward peak potential shifts towards positive potential showing that an irreversible electrode reaction takes place on the PdSn–MNT/C electrode surface.

**Fig. 7:**
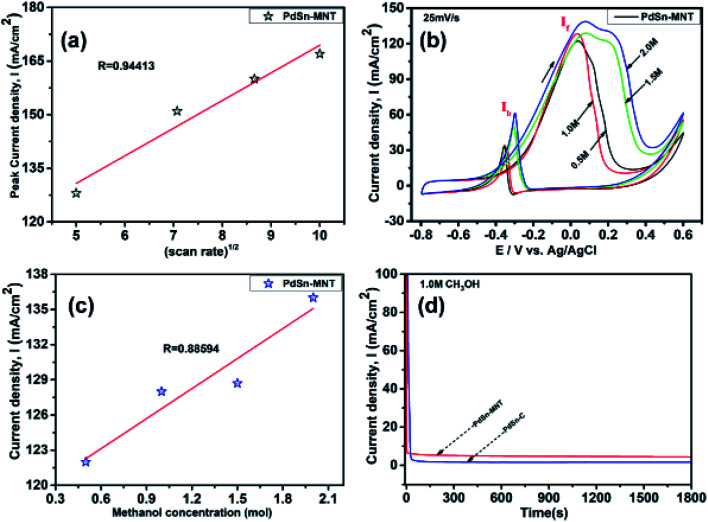
Cyclic voltammograms of nanostructured electrocatalyst (a) the square root of scan rate (b) CV of different concentrations of methanol from 0.5 M to 2.0 M at a scan rate 25 mV s^−1^ (c) square root of methanol concentration for PdSn–MNT/C and (d) chronoamperometry for PdSn–MNT/C and PdSn/C.

The oxidation current density and the square root of the scan rate had a linear relationship and it was shown in Fig. 7a which indicates that the electro-oxidation process proceeds as a diffusion-controlled reaction. Fig. 7b shows the typical CV for the PdSn–MNT/C electrodes as a function of methanol concentration between 0.5 and 2.0 M. The forward peak potential shifts slightly towards positive values with increasing methanol concentration. The corresponding peak current density was observed to increase linearly with the concentration of methanol as shown in Fig. 7c. Chronoamperometry and cyclic voltammetry are used to evaluate the stability of nanostructured PdSn–MNT/C and PdSn/C electrocatalyst for methanol oxidation, after fifty cycles are over. Fig. 7d shows the chronoamperometry curves for PdSn/C and PdSn–MNT/electrodes in 0.5 M KOH with one mole of methanol solution at a constant potential of 50 mV for 30 minutes. The current decayed rapidly with the PdSn/C electrode but slowly with the PdSn–MNT/C electrode. The PdSn–MNT/C electrode produces a higher current density than the PdSn/C electrode. These results also confirmed that the PdSn–MNT/C electrode has got greater electrocatalytic activity and higher stability than the PdSn/C.

The electrochemical surface area (ECSA) of an electrocatalyst reflects its intrinsic electrocatalytic activities, and the utilization ratios of noble metals are directly related to their dispersion and the ECSA. The hydrogen absorption into the bulk Pd lattice happens during the CV characterization process. The real surface area of Pd based catalyst has been calculated using hydrogen desorption curves. The ECSA values of the Pd-based electrocatalyst was calculated based on the following formula.
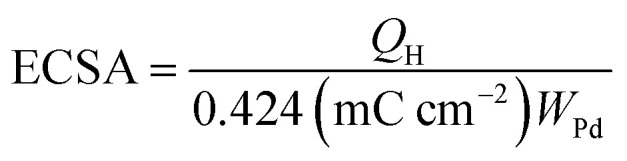


In the above formula, *W*_Pd_ gives the amount of Pd on the modified electrode. The calculated ECSA values were 60.00 m^2^ g^−1^ for PdSn/C and 152.44 m^2^ g^−1^ for PdSn–MNT/C. The fact that the ECSA value of the PdSn–MNT/C is higher than that of PdSn/C is due to the charge transfer interaction between Pd and Sn alters the electronic state of Pd atoms. It improves the electrocatalytic activity and stability of the PdSn–MNT/C.^42^ The synergetic effect of binary metal catalyst produces higher peak current density for the PdSN–MNT/C electrode. This experiment is in good accord with the published literature on other metal oxides promoted for the MOR.^43^ The metal oxide support electrocatalyst plays an essential role in oxidation reactions. The active surface oxygen atoms interact between the electrode and the support material that has enhanced the removal of CO poisoning in the course of methanol oxidation reactions.^44^ G. N. Vayssilov reported that the metal oxides have two types of interaction in the electrodes, such as the electron transfer from metal to metal oxide and the transfer of activated oxygen from metal oxide NiTiO_3_ to metal nanoparticles. The oxygen transfer through the metal and metal oxide boundary is a purely nanoscale effect.^45^

The present study was compared with the available literature and presented in Table 1. It clearly shows that PdSn–MNT/C is a promising electrocatalyst for methanol electro-oxidation in an alkaline environment for the fuel cells.

**Table 1:** Comparison of different electrode materials for methanol electro-oxidations

Electrode	*J* (mA cm^−2^)	Reference
Pd–Co/rGO/GCE	38.00	46
PdNPs–RGO	1.60	47
Pd–Ru/CoWO_4_/GNS	63.77	48
Pt_30_Pd_70_/C	33.0	49
PdSn/C	83.00	This work
PdSn–MNT/C	129.00	This work

#### 3.5.3 Polarization

**Fig. 8:**
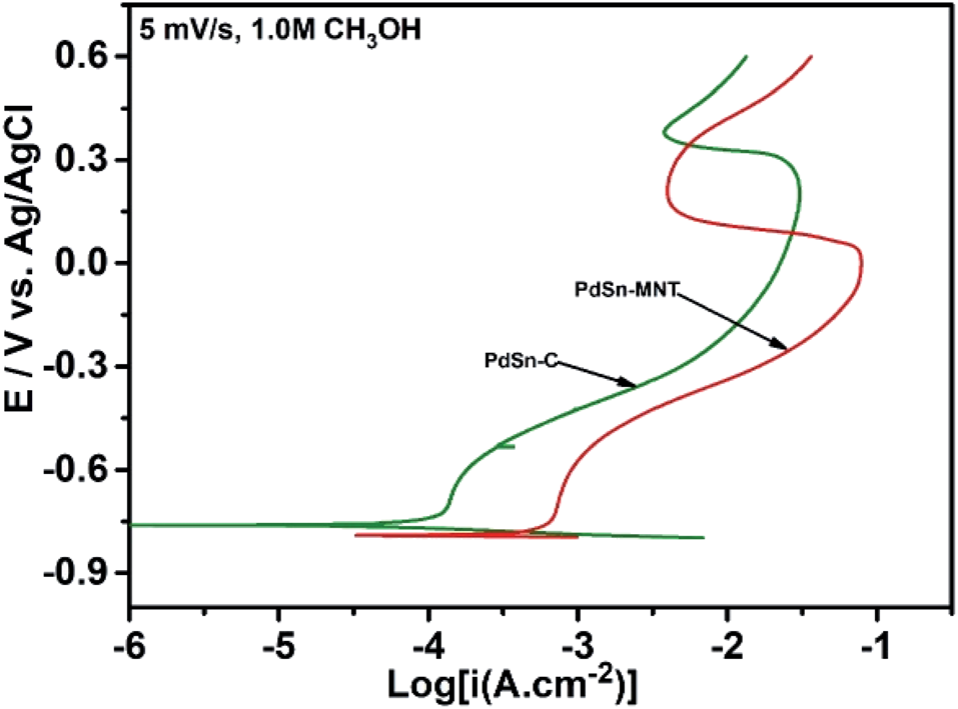
Tafel plots obtained from the electrocatalyst of this study at a scan rate of 5 mV s^−1^ in 0.5 M KOH is containing 1.0 M methanol.

The polarization measurements were carried out for PdSn/C and PdSn–Ni_1−*x*_Mn_*x*_TiO_3_/C in an alkaline medium with the presence of methanol to find the resistance of the electrocatalyst towards methanol oxidation. Tafel plots were fitted from the linear part of Polarization studies for PdSn/C and PdSn–MNT/C. The slope of the Tafel fits for PdSn/C and PdSn–MNT/C are found to be 178.0 and 171.0 mV dec^−1^ respectively. The Tafel plots are shown in Fig. 8, confirm that the interface of methanol and electrocatalyst is resulting in the formation of reactive intermediates. Tafel slope is experimental data that correlates the catalytic activity.^50^ It is reported that the smaller the value of the Tafel slope, the higher would be the electrocatalytic activity. The lesser magnitude of the Tafel slope indicates the higher tolerance of the active electrocatalyst towards the poisoning species.^51^ The results taken from this polarization experiment and the Tafel slope indicate that among the modified three electrodes PdSn–MNT/C glassy carbon electrode system has the lowest Tafel slope (Fig. 8). Therefore, PdSn–MNT/C exhibits a dynamic tolerance behaviour towards many intermediate species which is the important reason for its greater methanol electro-oxidation ability.

#### 3.5.4 Electrochemical impedance spectroscopy

**Fig. 9:**
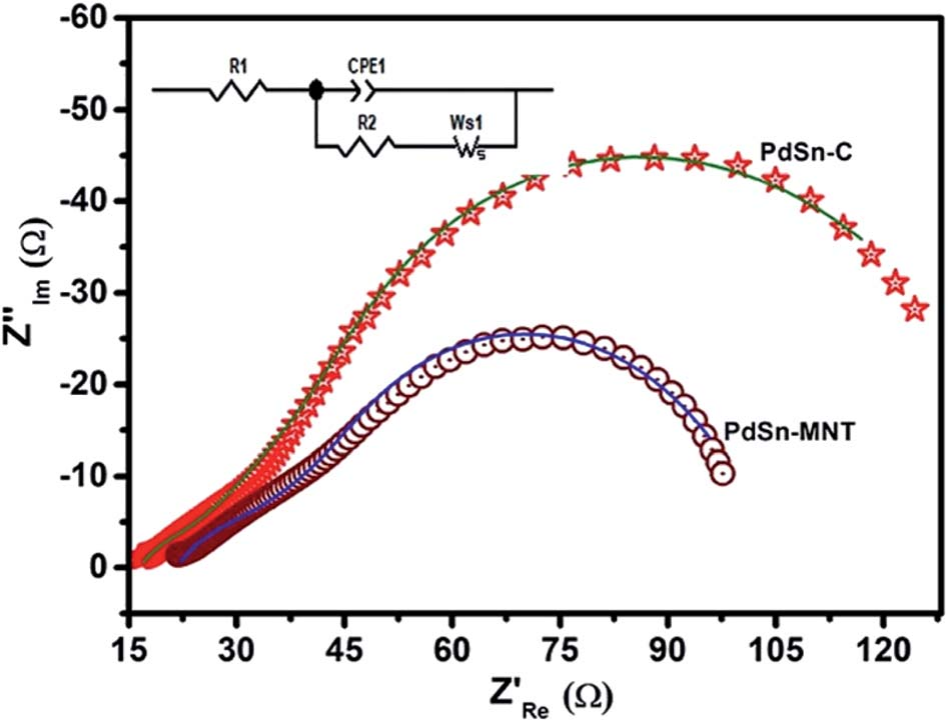
Electrochemical impedance (Nyquist) plots of PdSn–MNT/C and PdSn/C electrocatalyst with the equivalent circuit.

Electrochemical impedance spectroscopy has been widely used as a supporting method for cyclic voltammetry to investigate the charge transfer phenomena in the electrode–electrolyte interfacial properties of modified electrodes.^51,52^ The impedance behaviour of PdSn–MNT/C electrode was studied in 1.0 mol L^−1^ methanol with added 0.5 mol L^−1^ KOH. The equivalent circuit of PdSn–MNT/C and Nyquist plot of the impedance graph as depicted in Fig. 9 and the equivalent circuit also inserted in Fig. 9. To fit impedance spectra obtained on the PdSn–MNT/C electrode, *R*_1_, CPE, *R*_2_, and *W*, represents the solution resistance, constant phase elements, charge transfer resistance and Warburg elements that denotes a diffusion process, in the equivalent circuit.^53^ As demonstrated in Fig. 9 this charge transfer phenomenon is evaluated for different electrodes for methanol oxidation at 1.0 mol L^−1^ M^−1^ methanol. The charge transfer is high, and the resistance is low for PdSn–MNT/C compared to PdSn/C. This result is presented in Table 2, and it confirms that the rate of electro-oxidation of methanol increases. PdSn–MNT/C electrocatalyst was in good agreement with the cyclic voltammetry results.

**Table 2:** Fitted electrochemical impedance parameters based on the proposed equivalent circuit model as shown in the insert of Fig. 9

Electrode materials	*R*_1_ (Ω cm^−2^)	CPE-T (F cm^−2^)	CPE-P (Ω cm^−2^)	*R*_2_ (Ω cm^−2^)	*W* (Ω cm^2^ s ^−1/2^)
PdSn/C	19.6	0.00235	0.556	17.95	67.8
PdSn–MNT/C	18.49	0.00595	0.893	12.30	22.2

## 4. Conclusion

In summary, PdSn–MNT/C and PdSn/C prepared by microwave-assisted polyol method exhibited an enhanced electrocatalytic activity for the methanol oxidation reaction. XRD and TEM confirmed the phase stability of PdSn and the microscopic images ensured that PdSn was decorated over the metal oxide. Electrochemical studies using a cyclic voltammetry, polarization, chronoamperometry measurements revealed that the support of Mn-doped NiTiO_3_ for the PdSn electrocatalyst improved the electrocatalytic activity and stability in the course of methanol oxidation reactions. The methanol oxidation process that is carried out with the application of the Mn-doped NiTiO_3_–PdSn electrocatalyst was found to be an irreversible and diffusion-controlled electrode process. From the results, Mn-doped NiTiO_3_ support material for PdSn is a promising electrocatalyst for direct methanol oxidation in the alkaline environment for the fuel cells.

## Conflicts of interest

There are no conflicts to declare.

## Supplementary Material
